# *NtGNL1* Plays an Essential Role in Pollen Tube Tip Growth and Orientation Likely via Regulation of Post-Golgi Trafficking

**DOI:** 10.1371/journal.pone.0013401

**Published:** 2010-10-15

**Authors:** Fanglei Liao, Lu Wang, Li-Bo Yang, Xiongbo Peng, Mengxiang Sun

**Affiliations:** 1 Key Laboratory of Ministry of Education for Plant Developmental Biology, College of Life Sciences, Wuhan University, Wuhan, China; 2 College of Chemistry and Life Science, Zhejiang Normal University, Jinhua, China; 3 Biotechnology Department, Tobacco Research Institute of Chinese Academy of Agricultural Sciences, Qingdao, China; Iowa State University, United States of America

## Abstract

**Background:**

Tobacco GNOM LIKE 1 (*NtGNL1*), a new member of the Big/GBF family, is characterized by a sec 7 domain. Thus, we proposed that *NtGNL1* may function in regulating pollen tube growth for vesicle trafficking.

**Methodology/Principal Findings:**

To test this hypothesis, we used an RNAi technique to down-regulate *NtGNL1* expression and found that pollen tube growth and orientation were clearly inhibited. Cytological observations revealed that both timing and behavior of endocytosis was disrupted, and endosome trafficking to prevacuolar compartments (PVC) or multivesicular bodies (MVB) was altered in pollen tube tips. Moreover, NtGNL1 seemed to partially overlap with Golgi bodies, but clearly colocalized with putative late endosome compartments. We also observed that in such pollen tubes, the Golgi apparatus disassembled and fused with the endoplasmic reticulum, indicating abnormal post-Golgi trafficking. During this process, actin organization was also remodeled.

**Conclusions/Significance:**

Thus, we revealed that *NtGNL1* is essential for pollen tube growth and orientation and it likely functions via stabilizing the structure of the Golgi apparatus and ensuring post-Golgi trafficking.

## Introduction

During fertilization in higher plants, pollen landing on the receptive stigma of the pistil germinates, producing a pollen tube that grows and carries male gametes to the embryo sac for final gamete fusion [1]. Two basic processes take place during pollen tube growth, tube extension and orientation, which ensure that the pollen tube reaches the correct destination. The pollen tube extends via tip growth, during which polarized apical secretion results in unidirectional cell expansion [2]. This provides an excellent example of polarized growth and an ideal model system for clarifying the processes of organization and regulation [3], thus attracting broad interest for several decades [4]–[6]. Extensive studies have revealed that the dynamic extension and orientation process requires organization of the cell cytoskeleton [7], the deposition of the cell wall [8], and the balance of exocytosis and endocytosis [9]. Recently, a great deal of attention has been given to the latter case, specifically, vesicle trafficking at the tube apex [9]–[11].

Many vesicle-trafficking-related compartments have been characterized via electron microscopy (EM) [12]–[14] and co-localization with the endocytic marker FM4-64 [15]. The potential markers of endosome compartments were used to visualize vesicle trafficking vividly [16]. Living pollen tubes serve as an excellent research system to view endocytosis using FM4-64 [17]–[19]. Recently, a network of endocytosis and secretory pathways was constructed [20]. The trans-Golgi network (TGN) localizes in the center junction of these two pathways and associates with early endosome trafficking to regulate post-Golgi trafficking [20]–[21].

It was reported that large ARF-GEFs (ADP-Ribosylation Factor, Guanine-nucleotide Exchange Factor) were in charge of GDP-to-GTP exchange for ARF activation, which activated ARF-GTP interaction with some effectors to regulate vesicle trafficking [22]. Scientists only found ARF1 in Arabidopsis [23]. Several ARF-GEFs identified in Arabidopsis are known to regulate recycling endosomes, in addition to activating substrates. The first ARF-GEF isolated in plants was GNOM, which is sensitive to BFA and regulates PIN1 recycling between the plasma membrane and cytosol [24]. The function of GNOM has been extensively studied. It plays a critical role in apical–basal axis formation during early embryogenesis [25] and in the initiation of lateral root primordia by mediating auxin transport [26]. Detailed analysis revealed that GNOM is located in endosomes and regulates PIN1 polar distribution [27]. Recent studies have further revealed that GNOM is involved in transcytosis [28], indicating its critical role in the establishment of cell polarity.

GNOM-LIKE 1 (AtGNL1), a BFA-resistant ARF-GEF, carries out conventional functions, regulating vesicle trafficking between ER and Golgi bodies in *Arabidopsis* [29]–[30]. Phenotype analysis of a *gnl1* mutant revealed that competence in pollen tube competition was reduced. Additionally, AtGNL1 might be involved in PIN2 sorting to the vacuolar pathway [30]. Furthermore, BEN1 was also characterized as a new ARF-GEF family member to regulate early endosome trafficking in *Arabidopsis* using a fluorescence image-based screen [16]. These findings suggest that ARF-GEFs may play a critical role in regulating different stages of vesicle trafficking.

We have previously identified *NtGNL1* in tobacco, and our data indicated that its role, except in embryonic cell division pattern and root elongation, was, surprisingly, in pollen tube development [31]. Twisted pollen tubes and expanded tips of pollen tubes were observed in its RNAi lines. Pollen tube elongation was greatly inhibited, suggesting that *NtGNL1* anticipates its function in vesicle trafficking during pollen tube growth. As no genes in this family are reported to regulate pollen tube growth, we used an RNAi technique to down-regulate *NtGNL1* expression and confirmed that *NtGNL1* plays a critical role in pollen tube growth and orientation, and it likely functions via regulating post-Golgi trafficking.

## Results

### Inhibition of NtGNL1 expression disturbs pollen germination and pollen tube growth

**Figure 1:**
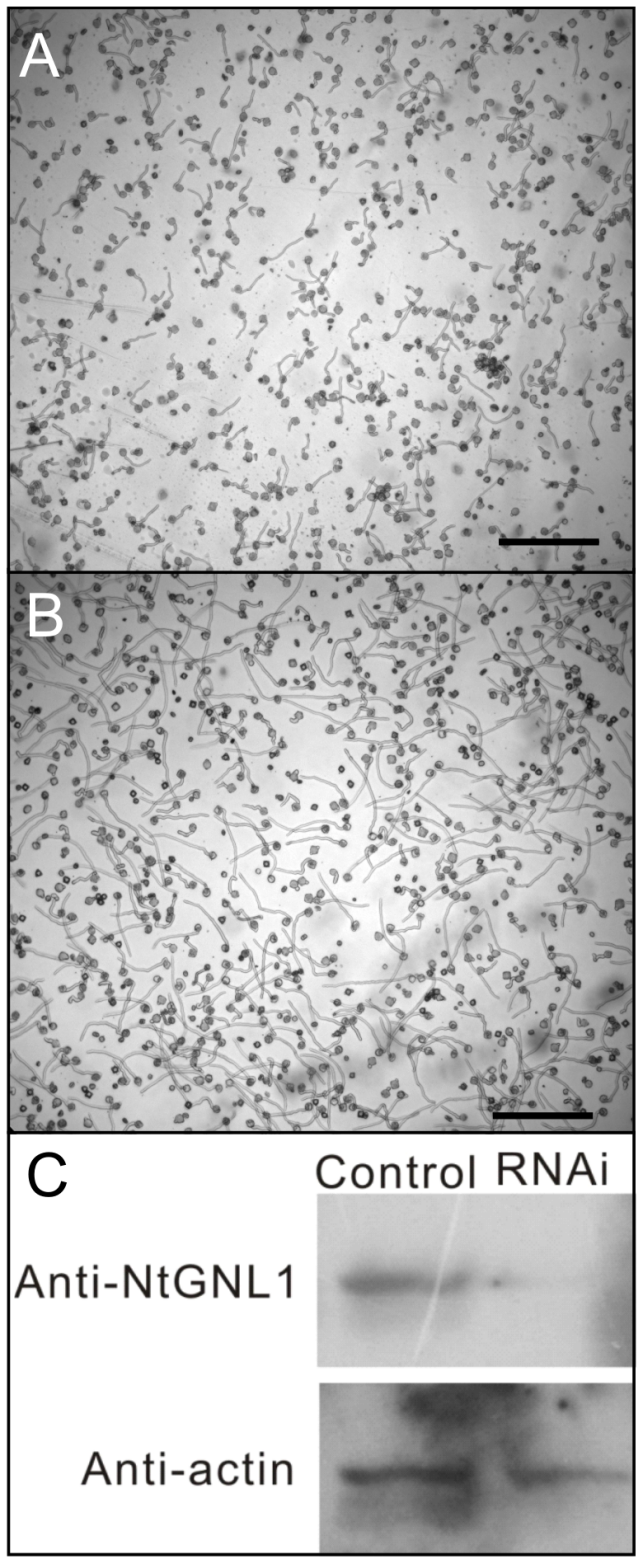
Pollen germination and pollen tube growth were inhibit in RNAi transgenic tobacco. A large population of pollen tubes of RNAi tobacco(A) compared with wild-type pollen tubes(B) to illustrate the difference of both length and number of pollen tubes; C: Western-blotting with anti-NtGNL1 to confirm the RNAi effects on pollen. NtGNL1 expression in RNAi lines. Anti-actin was used as an endogenous standard; wild-type SR1 was used as control. Bar = 100 µm.

We previously observed that in *NtGNL1* RNAi plants, both pollen tube growth and orientation were interrupted [31]. Given that no genes in this family have been found to play a role in pollen tube development, we further confirmed the phenotypes in RNAi plants. It was found that both germination frequency and pollen tube growth were greatly interrupted (Figure 1A, B), consistent with our previous results. We also further confirmed that *NtGNL1* was indeed down-regulated in RNAi pollen (Figure 1C).

### Temporal sequence of pollen tube FM4-64 uptake is disturbed by inhibiting *NtGNL1*

**Figure 2:**
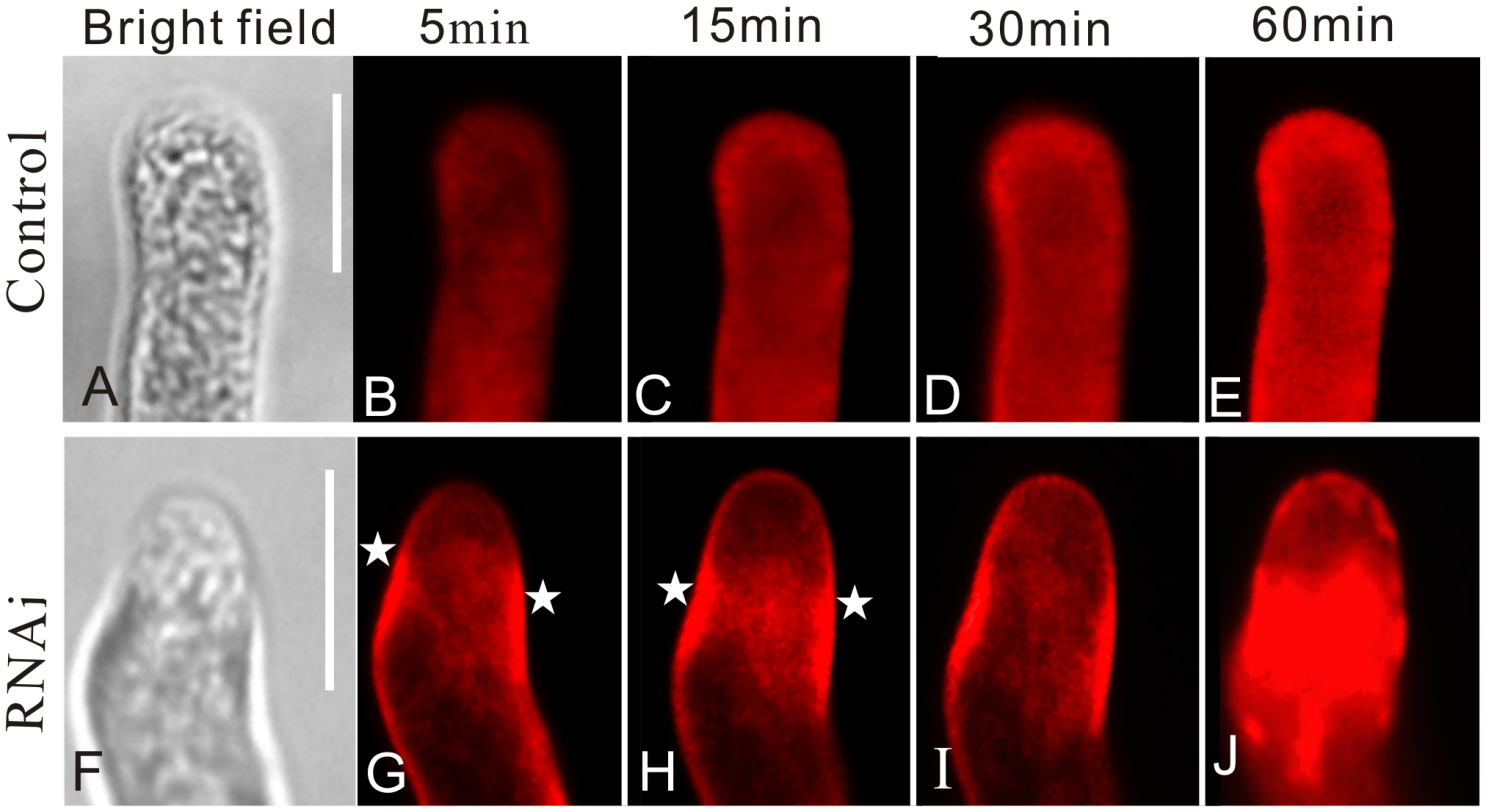
Inhibiting *NtGNL1* disturbed the temporal sequence of FM4-64 uptake in pollen tube. **A–E:** controls (wild-type pollen tubes) showing a strict time sequence of FM4-64 uptake with the fluorescence distributed evenly at the apex. **F–J:** RNAi transgenic pollen tubes. Stars indicate laggard vesicles. Pictures on the left lane are bright field images of corresponding pollen tubes. Pollen tubes were cultured for 3 h. Bar = 10 µm.

As reported previously, FM4-64 can trace endosome recycling at the apex of the pollen tube. FM4-64 entering the pollen tubes follows a strict temporal sequence from the plasma membrane to a population of small discrete internal structures, with subsequent appearance of the dye in the apical region and ultimately in vacuolar membranes [3]. To test whether abnormalities of pollen tube growth relate to vesicle trafficking, we compared the uptake process of FM4-64 in RNAi and normal samples to observe the dynamics of vesicle trafficking. In normal pollen tubes, the fluorescent signal of FM4-64 first appeared at the plasma membrane in the apex region (Figure 2A and B). Several minutes later, FM4-64 diffused to the interior apical region and displayed symmetry along the tip of the pollen tube (Figure 2C). This pattern remained for approximately 30 min (Figure 2D). Fluorescence spread to most parts of the pollen tube, remaining brightest in the apex (Figure 2E). However, RNAi pollen tubes grew in a zigzag pattern and displayed a different type of FM4-64 uptake. Specifically, when the pollen tubes began to turn, fluorescence appeared at the flank of the sub-apical region, not at the tip (Figure 2G). The distribution of the signal then gradually spread asymmetrically to the tip region (Figure 2H and I). Eventually, accumulated vesicles separated and moved backwards (Figure 2J). Clearly, diffusion of the FM4-64 signals was temporally disturbed in RNAi transgenic pollen tubes, and the time course and normal pace of endocytosis were inhibited due to down-regulation of *NtGNL1*.

### Down-regulation of *NtGNL1* Expression Results in an Altered Endosome Distribution Pattern

**Figure 3:**
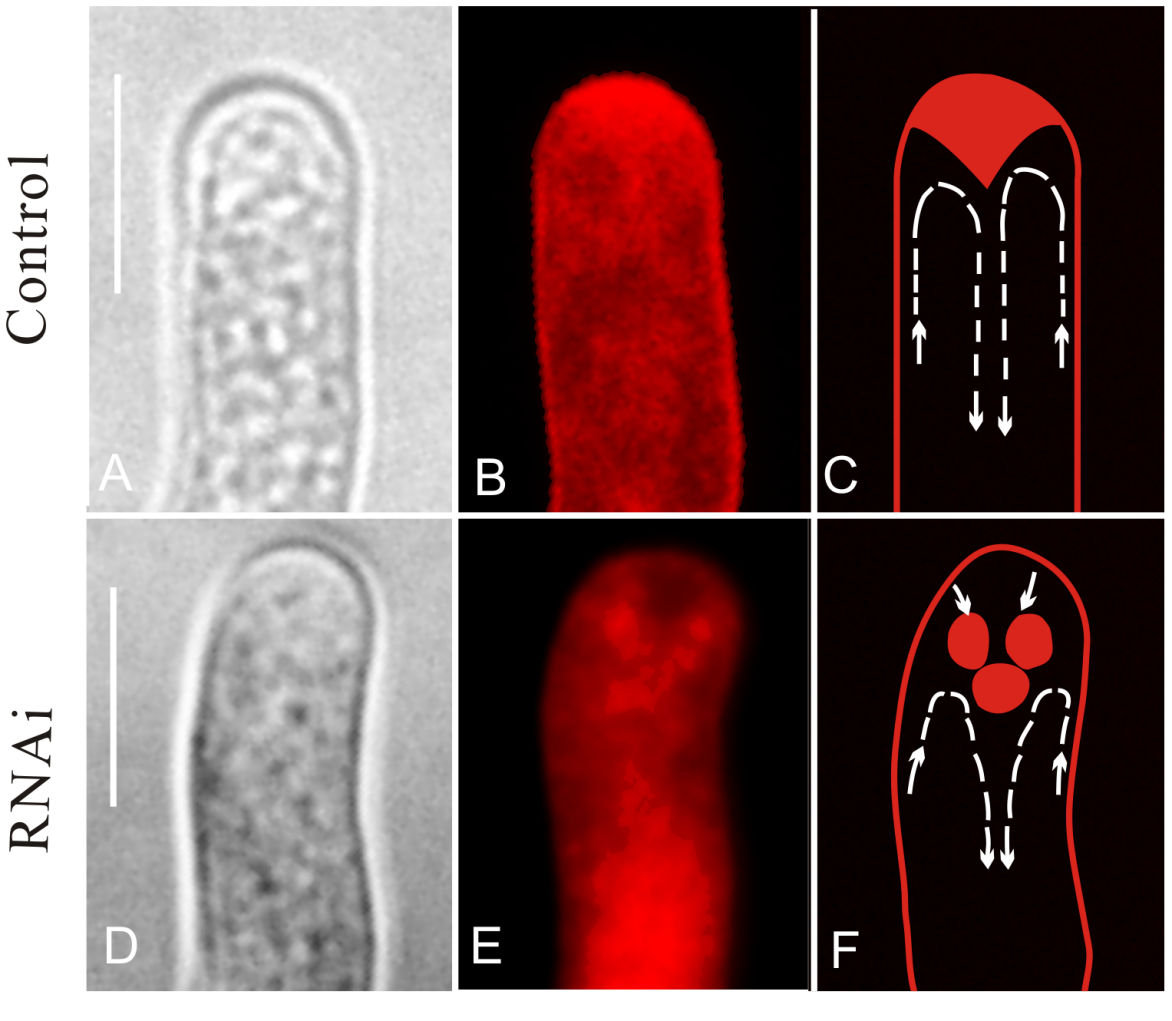
Distribution patterns of endosomes in RNAi pollen tubes were altered after down-regulating *NtGNL1* Expression. A and D are bright field images; others are fluorescent images or schematic diagrams. **A–C:** Control, showing normal fluorescence distribution in a converse V-shape. **D–F:** RNAi pollen tube showing fluorescence accumulated at the sub-apical region. The arrows indicate the direction of the vesicle movement. Images were taken between 30 and 60 min after loading FM4-64 to 3 h-cultured pollen tubes. Bar = 10 µm.

Following the application of FM4-64, we further compared endosome distribution patterns between the control and RNAi pollen tubes. In normal pollen tubes, fluorescence followed a symmetrical distribution, and a distinct gradient occurred at the tip. Typically, a reverse V-shaped distribution could be observed in control tubes (Figure 3A–C), whereas in RNAi pollen tubes, endosomes accumulated at the sub-apical region (Figure 3D–F). The endosome distribution pattern was obviously disturbed in these pollen tubes after the inhibition of *NtGNL1*expression. These results further demonstrate that *NtGNL1* is involved in regulating endosome trafficking, which is essential for normal pollen tube growth and orientation.

### Ultrastructural Observation Revealed Abnormal Post-Golgi Trafficking after *NtGNL1* Inhibition

**Figure 4:**
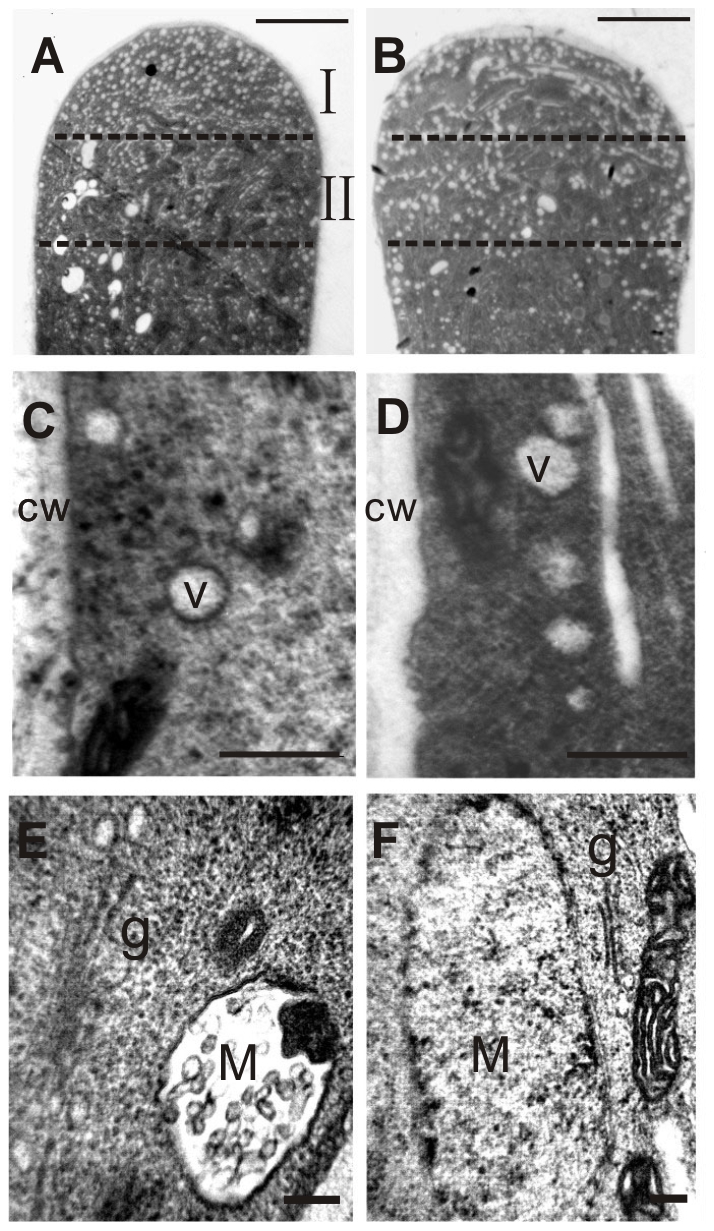
Ultrastructural observation of abnormal post-Golgi trafficking in RNAi pollen tubes. **A, C, E**: control (wild-type pollen). **B, D, F**: RNAi pollen tubes. **A, B**: Tip region of pollen tubes shows more small vesicles at the tip region I in control than in RNAi transgenic pollen tubes. Bar = 3 µm. **C, D:** more bigger vesicles at region II in RNAi transgenic than in control. Bar = 400 µm. E, F: Showing the volume of putative MVB in wild-type (E) and RNAi pollen tubes (F). Notice the comparative diameter of the putative PVCs/MVBs and adjacent Golgi bodies. Bar = 50 nm in (E); Bar = 100 nm in (F). cw: cell wall; g: Golgi bodies. M: putative PVCs/MVBs; V: vesicle.

Ultrastructural observations were carried out to examine vesicle transport and compartmentalization at the pollen apex and sub-apical region. The tip of the pollen tube was divided into two parts, region I (about 3 µm from the apex) and region II (3–6 µm from the apex) (Figure 4A, B). Compared with wild-type pollen tubes, RNAi pollen tubes were more vacuolated (Figure 4C, D). We mainly observed the vesicles in region II. The diameters of the vesicles in wild-type pollen tubes were usually less than 0.2 µm, but they were larger in RNAi pollen tubes (Table 1). Furthermore, the proportion of general vesicles (smaller than 0.2 µm) was reduced from 92.54% to 85.26% (Table 1), implying abnormal endosome cycling at the pollen tip.

**Table 1:** Various diameter of vesicles (%) compare in region II.

Material		Diameter(%)	
	<0.2 µm	0.2 µm–0.4 µm	>0.4 µm
**SR1**	**92.54**±1.08**	**2.63**±0.24 **	**4.83**±0.26**
**RNAi**	**85.26**±1.07**	**6.41**±0.09 **	**8.33**±0.36**

These vesicles were counted from 6 pollen tubes. n = 265±10. (P<0.01 [**]).

The size of the longest cistenae of Golgi stacks was proportional to the diameter of putative prevacuolar compartments/multivesicular bodies (PVCs/MVBs) in control pollen tubes (Figure 4E), whereas the putative PVCs/MVBs became much larger in RNAi pollen tubes (Figure 4F). This implies that vesicle trafficking might be disturbed in the transition of PVCs/MVBs to late endosomes or vacuoles.

**Figure 5:**
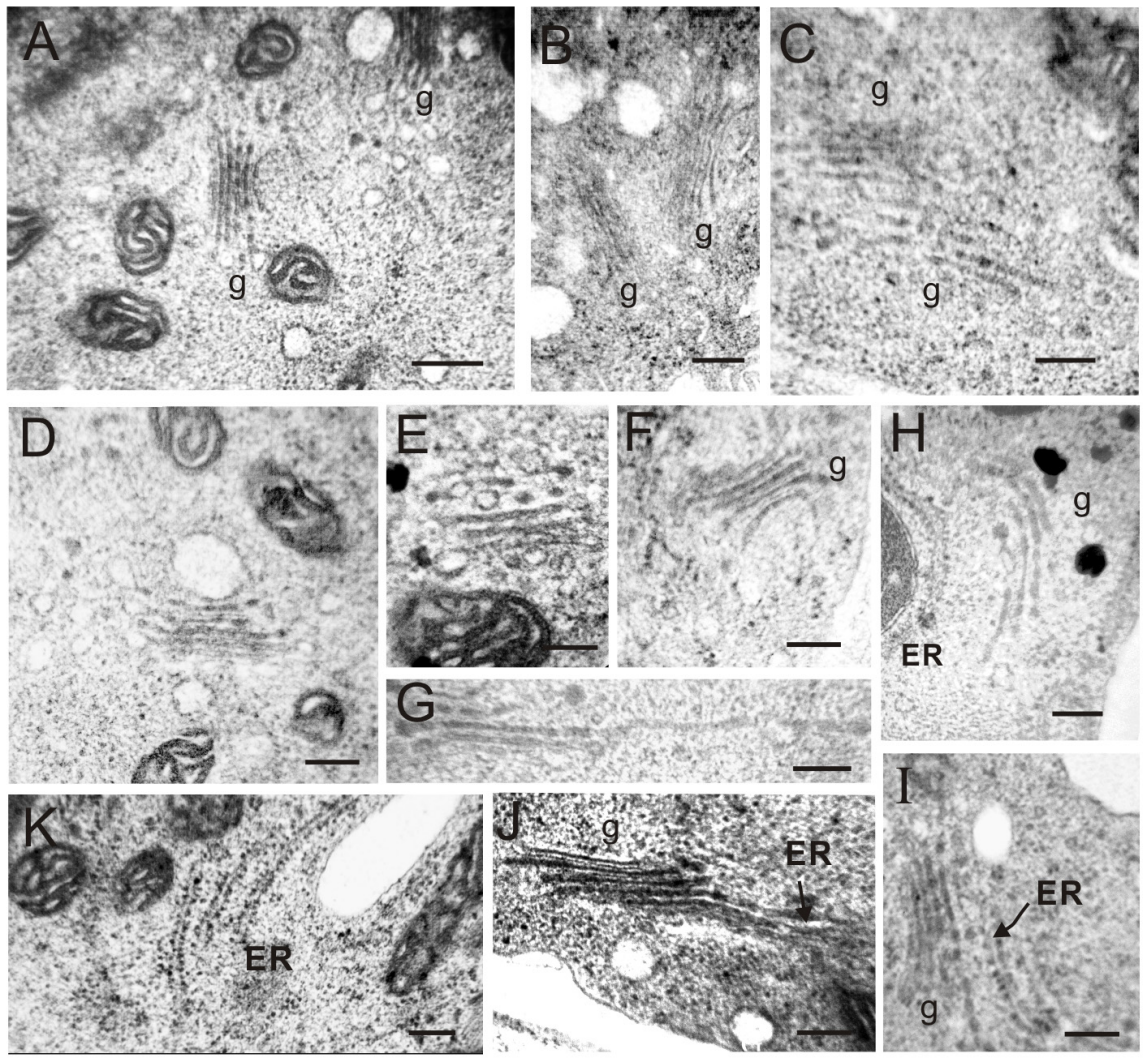
Various abnormal structures of Golgi apparatus in *NtGNL1* RNAi lines. **A:** Wide type pollen tube showing separated Golgi bodies. **B:** Accumulated Golgi bodies in RNAi pollen tubes. **C:** Segmentation of Golgi bodies. **D:** Wild type Golgi body. **E:** Normal Golgi apparatus in RNAi plants. **F:** Cisternal Golgi curved into a ring. **G:** Expanded and fragmented Golgi stacks. **H–J:** ER-Golgi hybrids. **K:** Normal ER in RNAi pollen tubes. Bar = 400 µm. ER: Endoplasmic reticulum; g: Golgi stacks. Bar = 200 µm.

The Golgi apparatus also exhibited a variety of abnormalities compared with wild-type (Figure 5A). Some Golgi stacks began to accumulate with PVCs (MVBs) in RNAi pollen tubes (Figure 5B), and some Golgi bodies showed piled up to each other or fragmentized (Figure 5C) compared with those in normal pollen tubes (Figure 5D) and with normal Golgi bodies in GNL1-down-regulated pollen tubes (Figure 5E). It was also observed that cis-Golgi curved into rings (Figure 5F) and expanded laterally (Figure 5G). This might reflect a prophase of Golgi-stack disassembly. We also observed that the Golgi apparatus began to fuse with the endoplasmic reticulum (ER) in pollen tubes (Figure 5H–J), a sign of Golgi structure collapse. So-called ER-Golgi hybrids in the pollen tubes of RNAi plants (Figure 5H–J) clearly suggested that vesicle trafficking between the ER and Golgi bodies was disrupted. In RNAi pollen tubes, about 50% of Golgi bodies showed abnormalities (Table 2). These results suggest that *NtGNL1* functions in the vesicle transport between the Golgi apparatus and PVC (MVB) or ER to maintain normal Golgi apparatus activity, indicating that post-Golgi trafficking is regulated by *NtGNL1*.

**Table 2:** Different Golgi bodies ratio (%) in RANi pollen tubes.

Golgi bodies (%)	Normal	Accumulation	Segmentation	Rings	Enlarging	ER-Golgi hybrids
**SR1**	**94.22**±2.69**	**6.90**±1.49**	**2.32**±**1.03**	**00.58**±1.01**	**1.14**±0.99**	0**
**RNAi**	**52.17**±1.37**	**13.04**±0.16**	**4.34**±**1.44**	**4.34**±0.93**	**17.39**±2.00**	**8.7**±0.30**

These vesicles were counted from 6 pollen tubes (n = 55±2). (P<0.01 [**]).

### NtGNL1 is Colocalized with Putative PVCs and Partially Overlapped with Golgi Bodies

**Figure 6:**
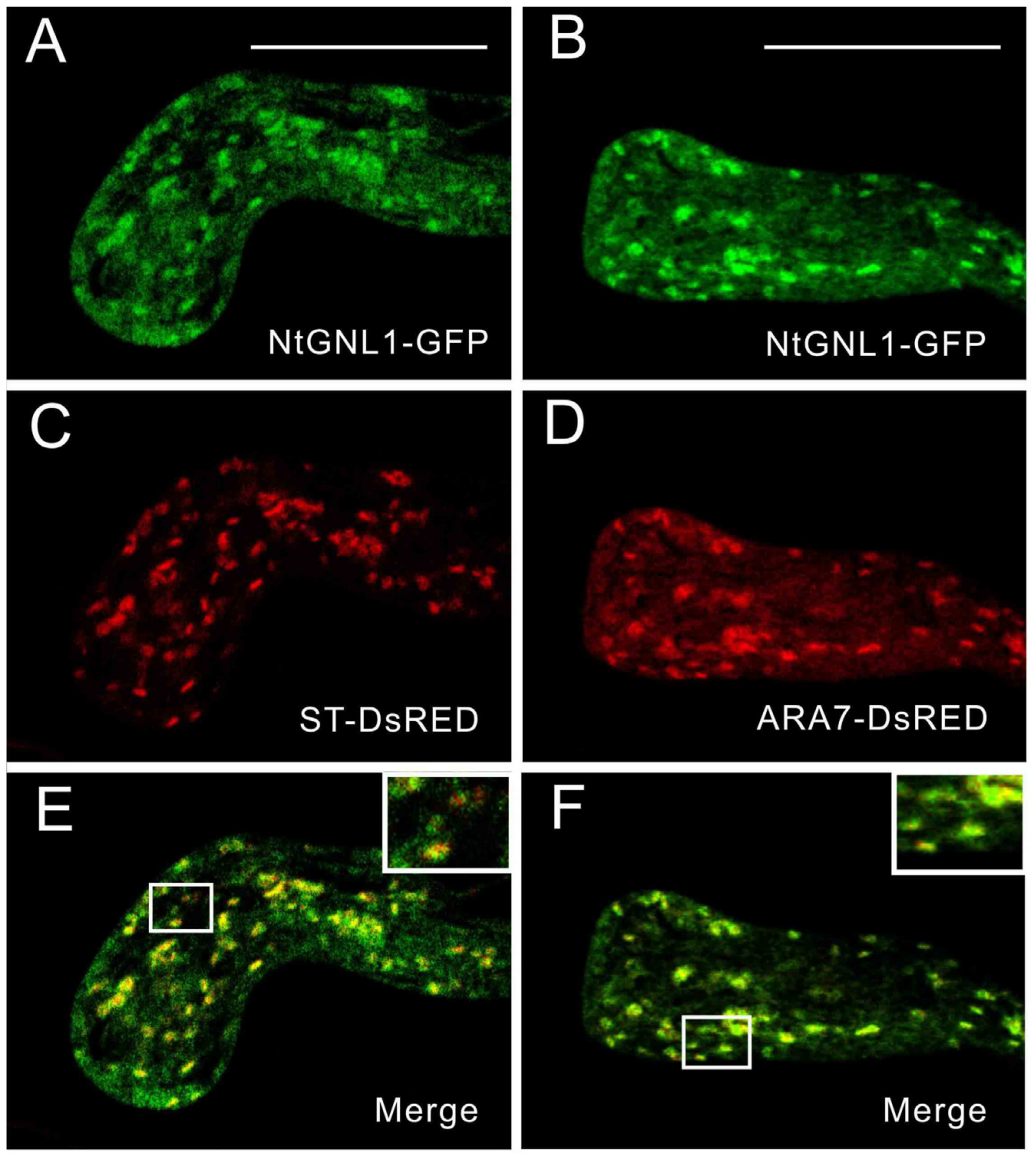
NtGNL1 colocalized with Golgi bodies and PVCs respectively. **A, B:** NtGNL1-GFP was transiently expressed in pollen tube. **C:** Transient expression of ST-DsRED. **D:** Transient expression of Ntrab5-DsRED. **E:** Merged image of A and C showing NtGNL1 and Golgi bodies partial colocalization. **F:** Merged image of B and D, showing NtGNL1 overlapped with PVCs. Bar = 20 µm.

To further confirm whether NtGNL1 is indeed located at Golgi bodies and vesicles, we constructed pACTIN11-ST-DsRED and pACTIN11-Ntrab5-DsRED to mark Golgi bodies and late endosomes (PVCs/MVBs), respectively. Each was transferred into tobacco pollen tubes together with pACTIN11-NtGNL1-GFP by a transient expression system (Figure 6). It is clear that NtGNL1 (Figure 6F, shown in green) was distributed in the pollen tube and colocalized with PVCs (Figure 6F,shown in red). However, it seemed partially overlapped with Golgi bodies (Figure 6E, shown in red). These results confirmed its critical role in the vesicle trafficking mentioned above. In both cases, NtGNL1 exhibited more extensive distribution than ST or Ntrab5 signals in the pollen tube, as expected.

### Actin Cytoskeleton is Disorganized after *NtGNL1* Inhibition

**Figure 7:**
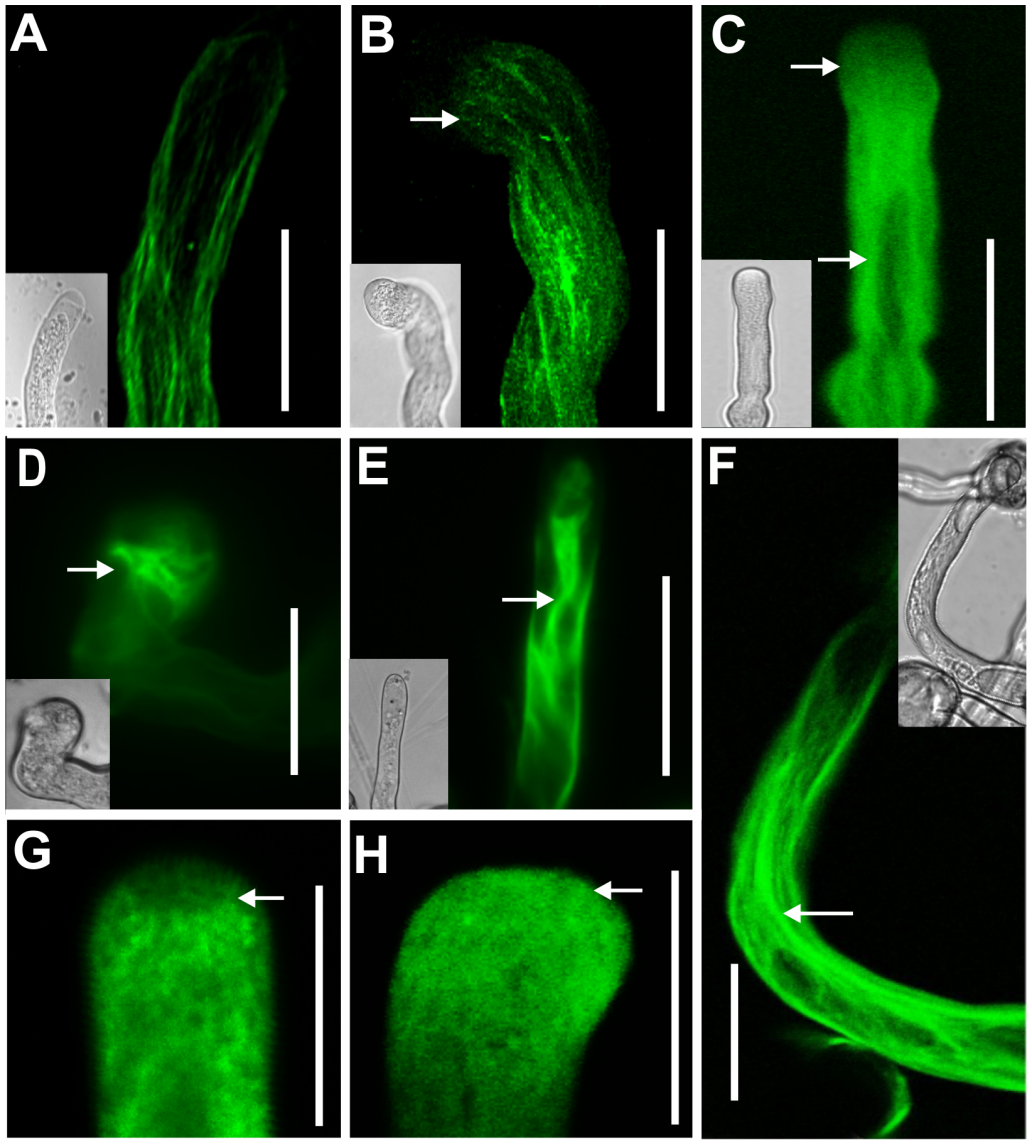
F-actin disorganization in *NtGNL1* RNAi pollen tubes. A and H: wild type pollen tubes. Others are RANi pollen tubes. **A**: Wild-type pollen tubes show no straight F-actin bundles at the clear zone. **B**: Twisted bundles extend to the clear zone of pollen tip(arrow). **C:** Condensed F-actin bundles (lower arrow) abundantly extend to the tip region of pollen tube (upper arrow). **D**: Thick bundles accumulate in expanded pollen tube tip and extend perpendicular to the tube long axis. **E**: Disorganized actin bundles accumulated at the pollen tip. **F**: Thick and twisted actin bundles bending in RNAi pollen tube. **G**: Actin fine fringe perpendicular to the plasma membrane (arrow) and absent at the clear zone in control. **H**: actin fringes collapsed at the sub-region and shifted to the clear zone (arrow). A- F. revealed by LAT52-ABD2-GFP; G and H were labeled by FITC–phalloidin. Bar = 20 µm in A- F. Bar = 10 µm in G and H.

Given that endosome movement in the pollen apical region may be actin dependent, we examined actin cytoskeleton organization in the *NtGNL1* expression-inhibited pollen tubes. By LAT52-promoted ABD2-GFP, we observed F-actin bundles in wild-type pollen tubes that were oriented parallel to the long axis of the pollen tubes (Figure 7A). However, these actin bundles did not extend to the clear zone of the pollen tubes (Figure 7A), which coincided with the typical model reported previously. Conversely, in RNAi pollen tubes, the F-actin cytoskeleton was obviously disorganized. Specifically, in the bulging, twisting pollen tubes, the actin bundles were thicker; they accumulated at the center of tubes and twisted and extended to the clear zone (Figure 7B–F). In bulging tips, these F-actin bundles even grew perpendicular to the long axis of the pollen tubes (Figure 7D), indicating loss of preprogrammed orientation. Some pollen tubes exhibited much thicker bundles and bends inside the RNAi pollen tubes, but fine structures were not observed in the tip region (Figure 7F).

To further elucidate the fine structure of F-actin in the tip region, particularly the fringe structure adjacent to the plasma membrane where endocytosis occurs, as well as in the clear zone where actin bundles normally terminate, we labeled the actin cytoskeleton with FITC–phalloidin in RNAi pollen tubes. In wild-type tubes, we observed the sub-apical cortical fringe structure, which was composed of short and fine actin bundles arranged in a parallel fashion perpendicular to the cell membrane (Figure 7G). At the clear zone of the pollen tube was a reverse V-shaped blank area, which coincided with the region occupied by a FM4-64-labeled vesicle organization of actins (Figure 5A). Conversely, most RNAi pollen tubes exhibited fine actin bundles within the clear zone that were arranged at random (Figure 7H) and shifted to the apex, which may be related to the disorientated vesicle trafficking previously observed in the pollen tip.

## Discussion

The phenotypes of the *NtGNL1* RNAi transgenic lines confirmed that *NtGNL1* indeed plays a critical role in regulating the polar extension of pollen tubes, the expected function of ARF-GEF in vesicle trafficking [24], [32]–[34]. Previous studies have reported that GNL1 regulates the formation of COPI-coated vesicles [29]. COPI-coated vesicles released from the Golgi apparatus accumulate by Brefeldin A (BFA) in mammalian cells [35], and a similar phenomenon was also observed in the tobacco BY-2 cells after BFA treatment [36]. This phenomenon suggested the existence of a BFA-sensitive ARF-GEF in tobacco. It was also reported that BFA treatment resulted in FM4-64 signals accumulating as small patches in tobacco pollen tubes [17]. In our experiments, RNAi pollen tubes showed abnormalities in the temporal sequence of FM4-64 uptake and its distribution pattern. FM4-64 signals were abnormally distributed as thick patches in the tip region of RNAi pollen tubes. This result indicates that the down-regulation of *NtGNL1* disrupted vesicle trafficking from the tip to the sub-region of pollen tubes and resulted in similar phenotypes as those observed after BFA inhibition.

As previously reported, BFA treatment of BY-2 cells produces BFA compartments of PVCs/MVBs as well as other endosomal compartments and also forms ER-Golgi hybrids [36]–[37]. In the present study, we confirmed that NtGNL1 partially colocalized with Golgi bodies and overlapped with PVCs in pollen tubes. PVCs/MVBs, as part of post-Golgi trafficking [20]–[21], play a role in vesicle trafficking between the plasma membrane and Golgi bodies and in regulating the retrograde vesicle transport from the tip to the sub-region of the pollen tube. Ultrastructural observations indicated that when NtGNL1 was down-regulated, more vacuolated vesicles appeared at the tip of the pollen tube, indicating that vesicle trafficking was blocked by PVCs/MVBs. The cisternae of the Golgi apparatus were reduced and expanded laterally. Different phases of cisternae of Golgi apparatus fragmentation could also be observed. Moreover, so-called ER-Golgi hybrids were formed in pollen tubes, which likely interrupted the recycling of COPI-coated vesicles. These data suggest that NtGNL1 plays a critical role in regulating vesicle trafficking as one of the possible BFA-sensitive ARF-GEF systems in tobacco pollen tubes. The data also suggest that *NtGNL1* may function by stabilizing the structure of the Golgi apparatus and maintaining COPI-coated vesicle recycling between the ER and Golgi apparatus. Based on these data, we assumed that the small vesicles from the Golgi apparatus were soon transformed to TGN or PVCs and thus, reduced their trafficking to the plasma membrane, which lowered the growth rate of the pollen tube and interrupted its orientation. By stabilizing the structure and function of the Golgi apparatus and maintaining properly oriented trafficking of early endosomes, *NtGNL1* contributes to the balance of endocytosis and secretary functions, and therefore maintains proper pollen tube polar extension. Recently, two distinct endocytic pathways were identified in tobacco pollen tubes [38]. Further work examining *NtGNL1* functions within distinct endosomal compartments may strengthen this proposal.

According to its sequence, NtGNL1 was predicted to be BFA sensitive in our previous report [31]. Therefore, it may be the target of BFA in pollen tubes. However, in *Arabidopsis*, GNL1 colocalized with Golgi bodies, but not with ARA7-labeled endosomes (PVCs) or FM4-64-labeled vesicles [29]. Furthermore, GNL1 was reported to be BFA resistant in *Arabidopsis* [29]. A recent report described that GNL1 serves a function in ER morphology in *Arabidopsis* [39]. Thus, NtGNL1 may function differently from AtGNL1 in the regulation of vesicle trafficking, at least in pollen tubes. Additionally, we found that actin organization was disturbed in *NtGNL1* down-regulated pollen tubes. There is no report on the role of this gene family in regulating actin dynamic. It might be interesting to know how abnormal vesicle trafficking could, in turn, influence actin organization.

## Materials and Methods

### Plant Materials

*Nicotiana tabacum* cv. Petite Havana SR1 plants were grown under 16 h of daylight at 25°C in a greenhouse or axenically in incubators. RNAi transgenic lines obtained from our previous work [31] were grown under the same conditions. Anthers were collected at room temperature to release pollen into the pollen germination medium (PGM).

### Pollen Germination and Pollen Tube Growth

Pollen was cultured in medium modified from Sun et al. [40]: 20% (w/v) sucrose, 0.01% (w/v) boric acid, 0.1 mM calcium chloride, 3 mM MES (methyl ester sulfonate), pH 5.6, incubated in dark at 25°C for 10 h.

### Antibody Preparation and Western Blot

NtGNL1 antibodies were prepared, and the Western blot for RNAi pollen was performed according to our previously published work [31].

### Plasmid Construction and Particle Bombardment-mediated Transient Expression in Tobacco Pollen

ABD2 was under the control of the pollen specific promoter LAT52 [41]. NtGNL1 (NCBI:EF520731), ST, and Ntrab5 (NCBI:X63875) are under the control of ACTIN11. The genomic fragment containing 796 bp upstream of the ACTIN11 start codon was PCR amplified as the ACTIN11 promoter. Amplified products were digested with EcoRI and KpnI and introduced upstream of GFP or DsRed (Clonetech, Mountain View, CA, USA) followed by the Nos polyadenylation sequence in the modified binary vector 1302 as plasmid of p068 and p067.

The tobacco Ntrab5 and GNL1 coding region was PCR amplified from cDNA of tobacco SR1 anther. The signal anchor sequences of rat sialyl transferase (ST) were PCR amplified from plasmid ST-GFP [42]. The GNL1-GFP construct was generated by inserting the GNL1 coding region into p068. The Ntrab5-DsRed and ST-DsRed constructs were generated by inserting the GNL1 coding region and ST sequence into p067, respectively.

Expression vectors were transferred into tobacco pollen grains by particle bombardment with a helium-driven particle accelerator (PDS-1000/He; Bio-Rad, Hercules, CA, USA) using methods modified from Fu et al. [43]. Mature pollen grains were collected form *N. tabacum* SR1, then suspended in PGM. A drop of 80–100 ml medium containing pollen grains from three flowers was applied to a piece of cellulose nitrate film (Optitran BA-S 85 Reinforced NC; Schleicher & Schuell, Dassel, Germany) placed on top of a piece of filter paper (Whatman, Florham Park, NJ, USA) in a Petri dish. Once liquid was drained from the membrane, an additional 1.5 ml PGM was added to the filter paper. Pollen grains were then immediately bombarded with DNA-coated gold particles using a PDS-1000/He particle delivery system (Bio-Rad). The microcarrier launch assembly was placed in the second slot from the top (level 2), and the target cells were placed at level 4 (the shortest distance from the stopping screen to the target cells). Rupture disks of 1,100 psi were chosen to accelerate macrocarriers under a vacuum of 28 inches of mercury. This was repeated three times for each Petri dish, and every firing required fresh plasmids. Gold particles (1.0 mm diameter) were coated with plasmid DNA according to the manufacturer's instructions (Bio-Rad) immediately before bombardment. Routinely, 1 mg particles were coated with 0.16 µg of pLAT52::ABD2 DNA. As control, the pLAT52 vector DNA was also bombarded. Bombarded pollen grains were incubated in a dark incubator for 30 min then washed into Petri dishes with 0.5 ml PGM. The pollen grains were left in the dark incubator for an additional 3–8 h before observation under an inverted microscope.

### Label of Actin Fringe in Pollen Tubes

A method modified from Xiang et al. [44] was used in this experiment. The germinated pollen was fixed in fixation buffer (4% paraformaldehyde, 50 mM PIPES, and 10% sucrose, pH 6.9) with vacuum infiltration for the first 5 min and then kept in the fixation buffer for 1 h. The fixed material was then washed in PIPES buffer (50 mM, pH 6.9) three times, followed by incubating in buffer (50 mM PIPES, 10% sucrose, 1% DMSO, and 0.01% Nonidet P-40, pH 6.9) with 60 nM rhodamine phalloidin (Molecular Probes, Carlsbad, CA, USA) for 1 h in the dark. The labeled pollen tubes were transferred onto a slide and visualized with a confocal laser scanning microscope (Leica SP2, Wetzlar, Germany).

### Microscopy

Pollen tubes were placed onto cover slips for microscopy (DMIRE 2; Leica, Wetzlar, Germany) using differential interference contrast (DIC). Images were taken with a cooled charge-coupled device (CCD) camera (microMAX; Roper Scientific-Princeton Instruments, Trenton, NJ, USA) using MetaMorph software. Pollen tube growth video imaging at the tip was performed at 1-h intervals after pollen germination using MetaMorph time lapse to acquire tip videos for 2 min at 2-s intervals. Pollen tube germination percentages and mean tube lengths were determined using Image J software (http://rsb.info.nih.gov/ij/). Loading of cells with FM4-64 dye in cover slips was generally achieved by application (at 4–8 mM) for 5 minutes after pollen tubes were cultured for 3 h. The pollen tubes were then centrifuged at 10,000 rpm to remove the dye and then transferred to slides.

Images were taken with a CCD or confocal microscope (Leica SP2).

### Ultrastructural Sectioning (EM)

For the ultrastructural examination, pollen tubes were fixed with paraformaldehyde (2%) and glutaraldehyde (0.1%) on ice (4°C) in PIPES buffer (50 mM, pH 6.8) for 2 h after thorough rinsing with cold buffer, then dehydrated in a graded ethanol series, including a post-fixation step with OsO_4_ (0.25%) in 30% ethanol overnight at 4°C, embedded in Spurr's resin. Samples were then transferred to room temperature, and finally polymerized at 45°C for 48 h. Ultrathin sections were post-stained with uranyl acetate/lead citrate before observation by EM.
